# Whole-Body Cryotherapy at −90 °C for 9 Weeks: Effects on Immune Function, Stress, and Immune-Related and Vascular Blood Parameters in Healthy Adults—Results of an Exploratory One-Armed Pilot Study

**DOI:** 10.3390/jcm15030967

**Published:** 2026-01-25

**Authors:** Punito Michael Aisenpreis, Sibylle Aisenpreis, Manuel Feisst, Robert Schleip

**Affiliations:** 1 ACT Akademie Murnau, 82418 Murnau, Germany; sib@somatic.de; 2 Institute of Medical Biometry, Heidelberg University, 69117 Heidelberg, Germany; feisst@imbi.uni-heidelberg.de; 3 Associate Professorship of Conservative and Rehabilitative Orthopaedics, TUM School of Medicine and Health, Technical University of Munich, 80333 München, Germany; robert.schleip@tum.de; 4 Department for Medical Professions, Diploma University of Applied Sciences, 37242 Bad Sooden-Allendorf, Germany

**Keywords:** whole-body cryotherapy, immune system, body composition, autonomic regulation, soluble ACE2, cytokines, stress

## Abstract

**Background/Objectives**: Whole-body cryotherapy (WBC), a brief exposure to extreme cold (−90 °C), has been proposed to modulate immune, metabolic, and stress-related pathways. This exploratory one-armed pilot study investigated the effects of an 18-session WBC protocol on immune markers, body composition, and perceived stress in healthy adults. **Methods**: Nineteen participants (mean age 52.9 ± 9.8 years) completed 18 WBC sessions over 9 weeks (3–6 min each), followed by a 9-week follow-up. Assessments were performed at baseline (M1), post-intervention (M2), and follow-up (M3). Primary outcomes included immune parameters (lymphocytes, granulocytes, cytokines, soluble ACE2), body composition (waist circumference, water compartments, lean mass), and perceived stress (Trier Inventory for Chronic Stress, TICS). **Results**: Waist circumference decreased from 83.8 ± 5.7 cm (M1) to 80.2 ± 4.2 cm (M2) (*p* = 0.001; M1 vs. M2; *p* = 0.004). Total body water (*p* = 0.008), lean body mass (*p* = 0.008), intracellular water (*p* = 0.005), and extracellular water (*p* = 0.021) also showed time-dependent effects. Immune modulation included increased lymphocytes (25.6 ± 7.1% to 29.3 ± 8.3%, *p* = 0.012) and decreased granulocytes (63.5 ± 6.8% to 58.7 ± 7.9%, *p* = 0.011) at M2. Anti-inflammatory IL-10 (virus-stimulated) rose markedly (33.5 ± 29.3 to 63.5 ± 50.5 pg/mL, *p* < 0.001), while IFN-γ (virus-stimulated) increased over time (*p* = 0.031). Soluble ACE2 decreased at follow-up (0.5 ± 0.7 to 0.3 ± 0.4 ng/mL, *p* = 0.029). Perceived stress improved in several TICS domains, including Work Overload (*p* = 0.009) and Pressure to Succeed (*p* = 0.018). **Conclusions**: This pilot study demonstrates that repeated WBC at −90 °C induces measurable changes in immune regulation, body composition, and perceived stress. These findings support the feasibility and potential physiological relevance of WBC and providing effect-size estimates for future randomized controlled trials.

## 1. Introduction

In recent years, increasing attention has been directed toward the human immune system and strategies to strengthen its function. A growing body of evidence indicates that physical inactivity, tobacco use, excessive alcohol consumption, diets high in ultra-processed foods, excessive intake of sugar and salt, insufficient sleep, and chronic psychological stress are associated with impaired immune function [1]. In contrast, regular physical activity, abstinence from tobacco and excessive alcohol consumption, consumption of minimally processed, nutritionally balanced diets, adequate sleep, and effective stress management have been linked to improved immune function and greater resilience to illness [2].

Beyond these lifestyle measures, Whole-Body cryotherapy (WBC)—the short-term therapeutic application (3–6 min) of extreme cold (approximately −90 °C)—has attracted increasing scientific and clinical interest. WBC was first developed in Japan around 40 years ago, and involves brief exposure in a cryochamber, during which participants perform light movements of the extremities and protect sensitive regions such as the ears, nose, and mouth. Initial studies in athletes reported reductions in inflammatory mediators and muscle enzymes [3]. Since then, WBC has demonstrated promising effects across multiple domains, including alleviation of chronic pain (e.g., fibromyalgia, osteoarthritis), facilitation of post-exercise recovery, improvements in sleep quality, mood, and reductions in depressive symptoms. Additional evidence points to potential benefits in weight management, autoimmune and neurological disorders, and even cognitive function [4,5,6].

More recently, mechanistic investigations have begun to clarify how WBC may influence immune and metabolic pathways. Clinical and experimental evidence suggests that WBC may modulate inflammatory cytokines, enhance adaptive immune responses, and contribute to improved metabolic flexibility and body composition [1,2,3,4,5,6,7,8,9]. WBC has also been explored in rehabilitation contexts, including post-COVID-19 recovery, refs. [10,11,12,13,14,15,16,17,18] where Soluble Angiotensin-Converting Enzyme 2 (sACE2) has been identified as a biomarker of disease severity [19,20,21,22,23,24,25].

Given this background, the present exploratory study investigated the effects of a standardized 9-week WBC program in middle-aged adults. Specifically, we focused on three domains: (1) Immune function, including Interferon-gamma (IFN-γ), Interleukin 2 (IL-2), Interleukin 10 (IL-10), lymphocyte subsets, tumor necrosis factor-alpha (TNF α)and plasma soluble ACE2 (sACE2); (2) body composition, assessed by bioelectrical impedance analysis (BIA), with a focus on waist circumference and lean/fat mass distribution; and (3) Psychological stress, measured by the validated Trier Inventory for Chronic Stress (TICS) [26,27,28,29]. This pilot study aimed to generate preliminary mechanistic data on the regulatory effects of WBC in humans and provide a foundation for the design of larger controlled trials [30,31,32,33,34,35,36,37,38].

## 2. Materials and Methods

### 2.1. Study Design

**Figure 1:**
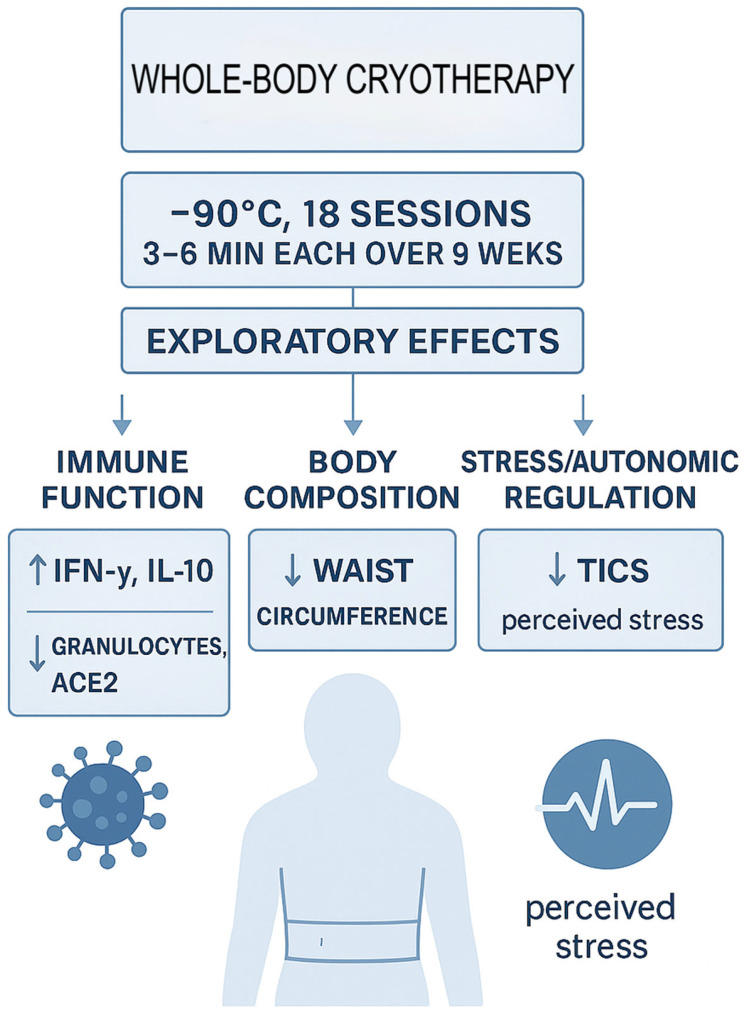
Schematic of the whole-body cryotherapy protocol (−90 °C, 18 sessions over 9 weeks) and principal observed effects on immune markers, sACE2, waist circumference, and stress. Arrows up: Increase, arrows down: Decrease.

The presented study was a one-armed prospective monocentric observational study including 20 adult participants. Patients before treatment were considered their own controls (Figure 1).

Participants underwent 18 sessions of cryotherapy over 9 weeks (−90 °C, 3–6 min each), followed by a 9-week post-intervention phase. Assessments were conducted at three time points: measurement 1 (baseline) before intervention, measurement 2 (post-intervention), and measurement 3 (follow-up). The measurements were performed exactly at the beginning and the end of the respective phase: at baseline directly before the first treatment, after 9 weeks directly after the last treatment and 9 weeks after the last treatment (follow-up visit). The measurement visits took place between 28 June 2023 and 23 December 2023.

### 2.2. Ethical Approval and Registration

The study was registered in the German Register for Clinical Trials (trial no. DRKS00031033 date 16 May 2023) after approval by the ethical committee of the Bayerische Landesärztekammer (https://ethikkommission.blaek.de/; approval number 22118; approval date 08 May 2023). All participants provided written informed consent.

### 2.3. Sample Size Justification

Since the present study is an exploratory monocentric study with a pilot character, no formal sample size planning was conducted. The number of cases of n = 20 was determined on the basis of available donations for the research and the limited capacities of a therapeutic practice for reasons of feasibility. The number of cases was considered sufficient to describe the continuous target variables collected in the study in such a way that initial insights into mechanisms of action of the intervention can be provided, and sample size calculations can be carried out for future confirmatory studies. In this context, it has been suggested that at least 20 participants represent a reasonable minimum for pilot studies based on statistical modelling considerations [39].

### 2.4. Recruitment

Participants were recruited in the corresponding author’s practice in Murnau via a local newspaper article, a local journal, and a video on a public website. Information was also disseminated to the Murnau health department as well as general practitioners and other health professionals in the Garmisch-Partenkirchen district in Bavaria, Germany.

### 2.5. Eligibility Criteria

The inclusion criteria were male/female/diverse, aged 40–75 years. The exclusion criteria for WBC in this study (to be clarified by preclinical medical diagnosis by the leading medical doctor of the study) comprised: pregnancy, severe hypertension (blood pressure > 180/100), acute or recent myocardial infarction, unstable angina, arrhythmia, symptomatic cardiovascular disease, pacemaker, peripheral arterial occlusive disease, venous thrombosis, acute or recent cerebrovascular accident, uncontrolled seizures, Raynaud’s syndrome, fever, tumor disease, symptomatic lung diseases, blood clotting disorders, severe anemia, infections, cold allergies, cold agglutinin disease and acute kidney and urinary tract diseases, as well as epileptic seizures. Further, increased intraocular pressure, such as glaucoma, represented an exclusion; for these patients, there is no exclusion of liability, including cases of unexplained increases in intraocular pressure [31]. Additional exclusion criteria included consumption of more than four cups of coffee per day, more than two alcoholic beverages per day, substantial dietary changes, and extreme sports, as well as other cold applications such as ice bathing and prior cryotherapy experience.

### 2.6. Procedures and Measurements

The procedure of the study went as follows: Before the intervention, there were measurements of Body composition (BIA), peripheral vascular activity, stress questionnaire, fascial tissue properties, and immune parameters via blood samples. The baseline measurement was followed by a 9-week program comprising 18 sessions (3–6 min) conducted in a Cryo.One whole-body cryotherapy chamber (Mecotec GmbH, Bitterfeld-Wolfen, Germany). After the intervention phase, a second measuring session was conducted following the same protocol as at baseline. After a subsequent 9-week post-intervention waiting period, a third measurement session was performed. Body composition was measured using bioelectrical impedance analysis (DATA Input Systems GmbH, Pöcking, Germany). Immune parameters included whole blood count analysis, CD4/CD8 subtyping (T cell Subsets (Helper/Cytotoxic)), and cytokine profiling under stimulated conditions, as well as measurement of plasma soluble ACE2 (sACE2). Stress perception was measured using the Trier Inventory for Chronic Stress (TICS), Hogrefe Verlag, Göttingen, Germany [26,27,28,29]. Peripheral vascular reactivity was assessed via blood volume pulse amplitude (BVP) with Nexus 10, Mindmedia B.V., Roermond, The Netherlands, Software Biotrace Version 2028A [32,33,40].

### 2.7. Laboratory Analyses

Laboratory analysis for sACE2 was performed using the Human sACE2 Enzyme-Linked Immunosorbent Assay (ELISA) kit (by Invitrogen (Thermo Fisher Scientific, Waltham, MA, USA). The serum samples from the participants, which had been frozen at −20 °C, were thawed at room temperature and diluted 1:2 with assay diluent from the test kit. This dilution level was chosen because preliminary tests showed that the 1:8 dilution specified by the test kit yielded in signals that were too low. Except for the changed dilutions, the ELISA was performed according to the test instructions, measured on the Tecan Reader Infinite F50 Plus, (Tecan, Maennedorf, Switzerland) and evaluated with the Tecan MagellanTM data analysis software (current version V7.5 Sunrise + at the time of the study). Laboratory analysis of sACE2, as well as Erythrocyte Sedimentation Rate (ESR), was conducted at the practice in Murnau.

All the other laboratory parameters were sent to and diagnosed by Lab4more Laboratory GmbH, Munich, Germany.

### 2.8. Protocol Deviations

During the study, it was discovered that one participant had had extensive cryotherapy exposure prior to enrollment; this participant was asked to withdraw and excluded from analysis.

### 2.9. Statistical Analysis

Patient cohort characteristics and clinical parameters were described using appropriate measures, depending on the scale: means and standard deviations for continuous variables medians with interquartile ranges, and total ranges for continuous variables; and absolute and relative frequencies for categorical variables. Repeated-measures analysis of variance (RM-ANOVA) was performed to analyze time courses of clinical parameters, which were illustrated using boxplots. Post hoc test of the RM-ANOVA was performed and adjusted by the Holm-Bonferroni method. Due to the exploratory character of this study, *p*-values were not adjusted for multiple testing (excluding post hoc tests), and no imputation of missing values was performed. Due to the relatively small sample size, no adjustment for confounding factors was considered in the statistical models. Furthermore, *p*-values have only descriptive meaning; *p*-values < 0.05 were determined as statistically significant, *p*-values < 0.1 were defined as trends. All analyses were performed in R version > 4.2.0.

## 3. Results

One participant experienced significant physical and mental strain due to family and work circumstances over the course of the study and became severely ill toward the end. This adverse event was deemed unrelated to the cryotherapy intervention. Consequently, the participant’s data was excluded from the final analysis.

### 3.1. Description of the Study Cohort and Received Cryotherapy

The detailed description of the study cohort and received cryotherapy can be found in Supplementary Table S1. The cohort consisted of 19 patients with an average age of 52.9 ± 9.8, and 4 (21%) male participants. The mean BMI was 23.8 (SD: 2.6). All participants received 18 sessions of cryotherapy with an average duration of 4.5 ± 0.76 min every 3.5 ± 0.4 days, indicating a consistent application of cryotherapy over all participants.

### 3.2. Bioimpedance Analysis

**Figure 2:**
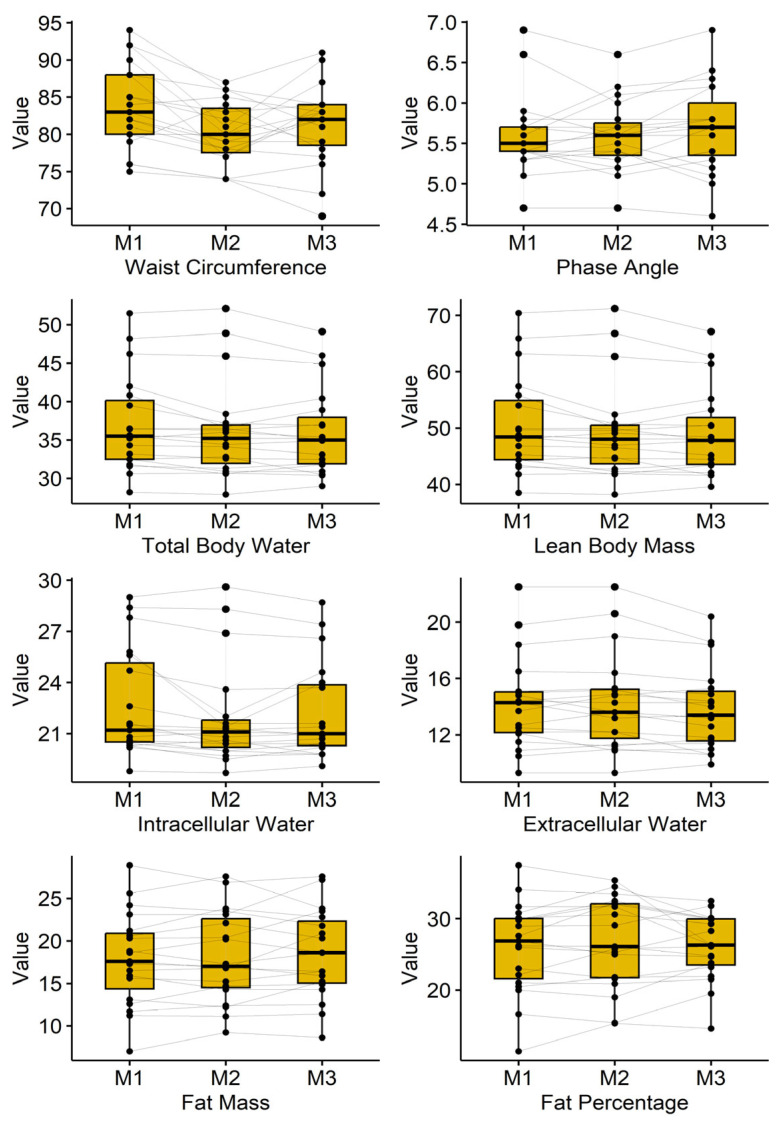
Boxplots and Participants’ courses of the bioimpedance analysis parameters at Measurement 1 (M1), Measurement 2 (M2), and Measurement 3 (M3).

Results of the bioimpedance analysis are presented in Table 1 and are illustrated in Figure 2. Waist circumference dropped from 83.8 ± 5.7 cm to 80.2 ± 4.2 cm and ended in 81.3 ± 5.5 cm (*p* = 0.001). Furthermore, total body water, lean body mass, as well as intercellular and extracellular water, showed a significant decrease.

### 3.3. Immuno-Blood Parameter and Further Blood Parameters

Table 2 shows the time course of blood parameters related to the immune system. Effects on Lymphocytes, Monocytes, and Granulocytes can be observed, especially directly after cryotherapy (Measurement 2); however, these effects dissipate by Measurement 3. Furthermore, the effects of cryotherapy on the virus pool can be seen: IL2 and INF-γ monotonically decrease and increase, respectively; IL10 shows a substantial impact at measurement 2. In general, effects are examined particularly from measurement 1 to measurement 2, with diminishing effects afterwards. Regarding the influence of cryotherapy on the further blood parameters ESR, sACE2, and BVP (mean and SD), no effects can be found for ESR, a significant decrease was observed for sACE2, surprisingly occurring mainly during follow-up (between measurement 2: 0.5 ± 0.7 and measurement 3: 0.3 ± 0.4; *p* = 0.029).

### 3.4. Subjective Perception of Stress

**Figure 3:**
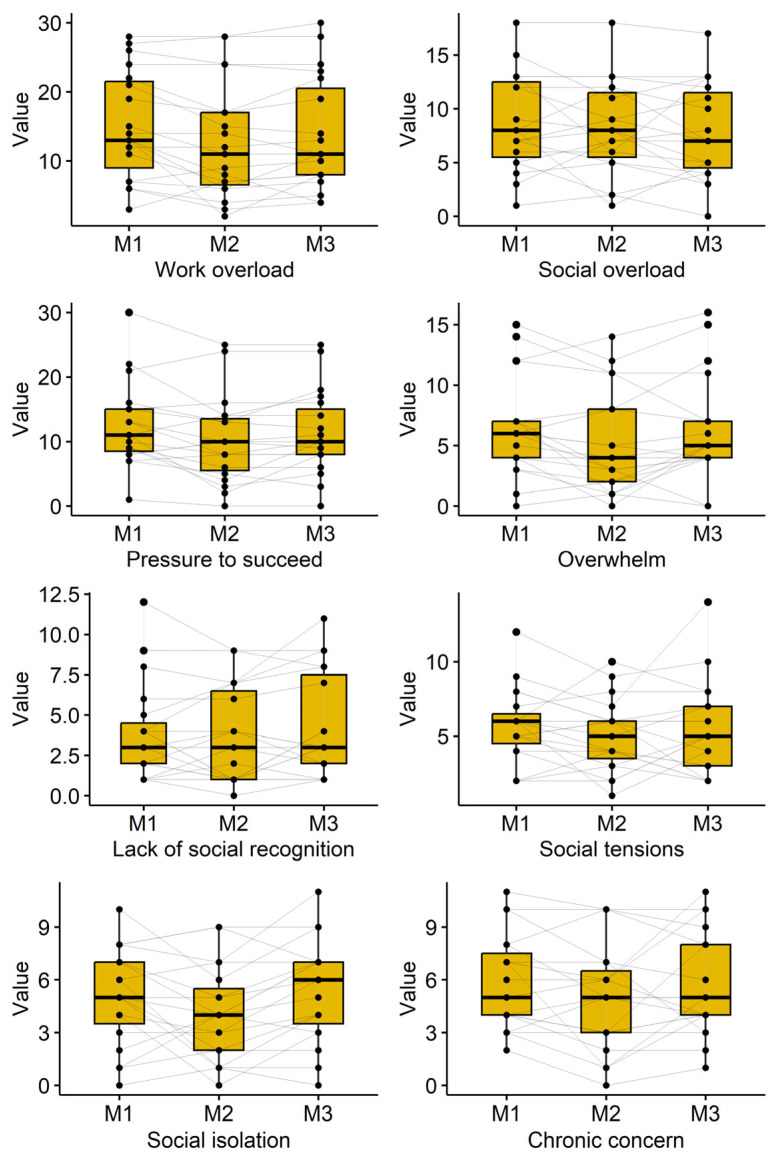
Boxplots and Participants’ courses of the Subjective perception of stress (TICS) parameters at Measurement 1 (M1), Measurement 2 (M2), and Measurement 3 (M3). Abbreviations: TICS—Trier Inventory of Chronic Stress.

Subjective stress perception was measured using the Trier Inventory for Chronic Stress (TICS). The course of the participant’s reported subjective stress perception is shown in Table 3 and Figure 3. Decreases were observed for the TICS subscales “work overload” (*p* = 0.009), “pressure to succeed” (*p* = 0.018), and “social isolation” (*p* = 0.049). A reduction was also observed for the scale “overwhelm” directly after cryotherapy (6.4 ± 4.1 to 5.2 ± 4.3; *p* = 0.081), but values returned towards baseline at follow-up (6.2 ± 4.4). Several other TICS subscales showed reduced scores immediately after cryotherapy, which often attenuated at follow-up.

## 4. Discussion

This exploratory pilot study demonstrates that a structured 9-week program of whole-body cryotherapy (WBC) at −90 °C followed by a 9-week observation period is associated with measurable systemic changes in immune function, body composition, and vascular regulation. Since this study is a single-arm pilot study in which participants served as their own pre-post controls, the findings should be interpreted with appropriate caution.

The most consistent findings were a reduction in waist circumference, an increase in lymphocytes and interferon-γ, a rise in IL-10, a decline in granulocytes, and a significant decrease in plasma soluble ACE2 (sACE2). Together, these results suggest that repeated WBC exposure may exert immunomodulatory and metabolic effects relevant to health maintenance and disease prevention.

Immune modulation: The observed increase in interferon-γ and IL-10, alongside a decline in granulocytes, indicates a shift toward a more balanced immune phenotype, consistent with literature showing cryotherapy-induced regulation of cytokine networks and leukocyte subsets [6,7,8,9,10]. The anti-inflammatory signal of IL-10, although modest, may support a regulatory immune state, while stable or slightly reduced pro-inflammatory mediators align with earlier cold exposure findings [11,12,13]. These results add to evidence that WBC may attenuate low-grade inflammation and improve immune surveillance in middle-aged adults [24,25,37,38].

Soluble ACE2: The reduction in plasma sACE2 is of particular interest. Beyond its role as a receptor in SARS-CoV-2 infection, sACE2 is recognized as a regulator of vascular and metabolic homeostasis. Lower circulating sACE2 after WBC may reflect an adaptive endothelial or neurohumoral response, possibly mediated by catecholamine release or nitric oxide signaling. This effect has not been reported in prior WBC trials, underscoring the novelty of our protocol and its implications for vascular–immune crosstalk. Nevertheless, interpretation must remain cautious: hydration, diet, and inter-individual variability may confound sACE2 dynamics; controlled studies are needed to validate this signal.

Body composition: We observed a reduction in waist circumference without significant changes in total fat or lean mass. This may reflect regional adaptation, redistribution of body water, or subtle changes in visceral adiposity not detectable by bioimpedance.

We recognize that the lack of a control group is a significant weakness of this study; thus, the results should be read as preliminary and interpreted with care. With that in mind, the BIA data suggest a possible—though statistically non-significant—trend toward a more balanced distribution of body water between fat and lean tissue compartments. Many participants showed mild signs consistent with edema before the WBC sessions, and across the intervention, their readings moved toward lower water values and a profile the device interprets as more balanced hydration.

To date, we are not aware of published work directly linking WBC to hydration status. This means other explanations cannot be ruled out. In particular, the observed changes might reflect a general dehydration effect rather than a specific normalization of fluid distribution. We also did not track participants’ diet or fluid intake during the study, which limits what we can conclude about mechanisms. Still, using the manufacturer’s reference framework for the BIA system, the post-WBC patterns are classified as indicating improved hydration balance. We see these findings mainly as hypothesis-generating and hope they encourage future controlled studies that include careful monitoring of fluid intake and additional hydration measures. Future studies should incorporate imaging modalities such as Dual-Energy-Xray Absorptiometry (DXA) or Magnetic Resonance Imaging (MRI), as well as fluid intake questionnaires for the participants to clarify these findings.

Stress and adaptation: Although modest, stress perception measured by TICS, declined post-intervention, aligning with reports that WBC may support psychological resilience, sleep quality, and mood [14,15,26,27,28,29]. Acute changes in vascular reactivity observed during the intervention phase (e.g., transient increases in BVP amplitude) [40] suggest stimulation of autonomic pathways, possibly linked to norepinephrine surges during cold exposure. However, these responses were not sustained, reinforcing that WBC’s primary benefits may lie in immune and metabolic modulation rather than durable autonomic changes.

Age and sex considerations: Our cohort (40–75 years, 79% female) differs from most WBC studies that focus on younger male athletes. Aging is associated with diminished mitochondrial plasticity, altered immune remodeling, and a senescence-associated secretory phenotype, all of which may blunt cytokine responses to interventions. This may explain the more modest cytokine shifts observed here compared to younger cohorts. Sex differences in cold adaptation and immune regulation are well documented, but could not be examined in this small, unbalanced sample. Larger, sex-stratified studies are warranted.

Comparison with existing literature: Our findings are consistent with reports that cryotherapy reduces IL-6, TNF-α, and CRP, improves recovery, and reduces stress perception [3,6,7,8,9,10,11,12,13,14,15,16]. Unlike protocols using −110 °C or cold-water immersion, we employed standardized −90 °C cold-air exposure without mechanical confounders, enabling clearer attribution to thermal stress. The consistent 18-session program over 9 weeks adds to the limited data on longer-term adaptations in non-athletic, middle-aged populations.

Exploratory parameters such as myofascial stiffness, joint flexibility (finger-to-floor distance), and mechanical pain sensitivity (algometry) were assessed but are not reported in detail due to limited sample sizes and non-prespecified status. These measures will be targeted for standardized collection in future confirmatory trials.

Limitations: This study is limited by its small sample size and lack of a control group, which restricts causal inference. The sex imbalance (79% female) limits generalizability. Hydration and dietary intake were not standardized, which confounded the interpretation of bioimpedance outcomes. Subgroup analyses (e.g., stiffness, algometry) were underpowered and are not reported here. While preliminary signals on sACE2 and cytokines are intriguing, they require validation in larger randomized controlled trials.

Implications: Despite limitations, the reduction in sACE2 alongside immune modulation and improved central adiposity suggests potential roles for WBC in preventive medicine and metabolic health. Given safety and feasibility, larger controlled studies are justified to define therapeutic potential and optimize protocols for specific populations, particularly older adults.

## 5. Conclusions

Our findings demonstrate that whole-body cryotherapy at −90 °C induces measurable and systemic physiological adaptations, most prominently a reduction in inflammatory markers, improvements in body composition parameters such as waist circumference, and modulation of immune regulation. By applying a uniform cold stimulus without mechanical confounders, this protocol reveals subtle but reproducible adaptation mechanisms, offering new insights into how the human body responds to repeated thermal stress [41].

These results highlight that WBC acts on multiple regulatory levels—including hydration, immune function, and vascular balance—extending beyond the traditionally reported effects of pain relief or local inflammation. The observed decrease in soluble ACE2 is of particular relevance, as it indicates a potential modulation of vascular–immune crosstalk and may represent a protective adjustment in metabolic and stress-related pathways.

Collectively, this study underscores the complex, multilevel impact of WBC and the need for system-level frameworks that can capture non-linear, time-sensitive, and individualized responses to cryotherapy. While preliminary, these findings suggest that WBC may serve as a viable non-pharmacological adjunct in metabolic regulation, stress resilience, and immune support.

This investigation did not include a proper control group. Instead, participants’ baseline health status was used as a within-subject reference for the intervention period. Although unlikely, it cannot be ruled out that comparable improvements might have occurred even without the intervention, for example, due to concurrent lifestyle changes or spontaneous remission. Future clinical trials should build on the encouraging findings of this pilot study and re-examine these effects in appropriately larger, controlled, randomized trials, exploring sex-specific responses and investigating tailored cryotherapy protocols to optimize long-term outcomes.

## Figures and Tables

**Table 1:** Bioimpedance analysis parameters. RM-ANOVA and post-hoc tests (adjusted by Holm-Bonferroni method).

Parameter	Measurement 1 (Mean ± SD)	Measurement 2 (Mean ± SD)	Measurement 3 (Mean ± SD)	*p*-Value Anova	M1 vs. M2	M1 vs. M3	M2 vs. M3
Waist Circumference (cm)	83.8 ± 5.7	80.2 ± 4.2	81.3 ± 5.5	**0.001**	**0.004**	**0.008**	0.316
Phase Angle	5.60 ± 0.49	5.60 ± 0.43	5.69 ± 0.55	0.240	1.000	0.374	0.360
Total Body Water (L)	36.95 ± 6.3	36.24 ± 6.4	35.99 ± 5.7	**0.008**	0.067	**0.003**	0.483
Lean Body Mass (kg)	50.48 ± 8.6	49.49 ± 8.7	49.17 ± 7.7	**0.008**	0.063	**0.004**	0.497
Intracellular Water (L)	22.71 ± 3.2	21.95 ± 3.0	22.16 ± 2.8	**0.005**	**0.035**	**0.002**	0.347
Extracellular Water (L)	14.24 ± 3.3	14.29 ± 3.4	13.81 ± 2.9	**0.021**	0.760	0.098	**0.050**
Fat Mass (kg)	17.88 ± 5.5	18.14 ± 5.4	18.46 ± 5.2	0.396	0.882	0.597	0.882
Fat Percentage (%)	26.0 ± 6.4	26.7 ± 6.4	26.0 ± 4.6	0.530	0.486	0.98	0.762

Bold means statistically significant changes.

**Table 2:** Immuno-blood parameter across time points. RM-ANOVA and post-hoc tests (adjusted by Holm-Bonferroni method).

Parameter	Measurement 1 (Mean ± SD)	Measurement 2 (Mean ± SD)	Measurement 3 (Mean ± SD)	*p*-Value Anova	M1 vs. M2	M1 vs. M3	M2 vs. M3
Lymphocytes	25.6 ± 7.1	29.3 ± 8.3	26.9 ± 6.9	**0.012**	**0.016**	0.467	0.079
Monocytes	7.9 ± 1.4	8.5 ± 2.4	7.8 ± 1.5	0.121	0.45	0.783	0.486
Granulocytes	63.5 ± 6.8	58.7 ± 7.9	62.2 ± 6.6	**0.011**	**0.017**	0.390	0.102
T-cells	72.6 ± 8.5	72.7 ± 8.2	72.7 ± 7.7	0.986	1.000	1.000	1.000
CD4	45.5 ± 9.3	45.4 ± 9.5	45.7 ± 9.1	0.982	1.000	1.000	1.000
CD8	23.8 ± 7.5	22.9 ± 9.5	23.8 ± 7.8	0.503	1.000	1.000	1.000
CD4/CD8 ratio	2.2 ± 1.0	2.2 ± 1.2	2.2 ± 1.2	0.799	1.000	1.000	1.000
IL2-virus	36.4 ± 29.3	28.5 ± 25.2	22.9 ± 26.7	0.069	0.394	0.101	0.394
IL10-virus	33.5 ± 29.3	63.5 ± 50.5	20.5 ± 26.8	**<0.001**	**0.011**	**0.015**	**0.001**
INF-γ-virus	18.9 ± 14.8	19.4 ± 18.7	44 ± 62.1	**0.031**	0.895	0.178	0.178
TNF-α-virus	760 ± 830	795 ± 764	620 ± 965	0.681	1.000	1.000	1.000
IL2-bacteria	63.8 ± 158	24.1 ± 32.3	27.6 ± 61.1	0.169	0.453	0.453	0.683
IL10-bacteria	5.5 ± 8.8	17.3 ± 34.1	3.6 ± 10.9	0.092	0.345	0.537	0.345
INF-γ-bacteria	1.6 ± 3.3	1.7 ± 4.8	2.6 ± 5.5	0.400	0.893	0.622	0.435
TNF-α-bacteria	6.9 ± 9.2	8.6 ± 15.4	6.9 ± 9.5	0.799	1.000	1.000	1.000
IL2-fungal	10.6 ± 15.1	6.2 ± 5.7	4.4 ± 4.6	0.063	0.184	0.184	0.184
IL10-fungal	53.5 ± 40.0	73.1 ± 51.7	34.9 ± 39.6	**0.001**	0.068	**0.033**	**0.007**
INF-γ-fungal	2.1 ± 4.8	2.2 ± 5.9	1.8 ± 3.5	0.936	1.000	1.000	1.000
TNF-α-fungal	577 ± 675	696 ± 436	790 ± 296	0.711	1.000	1.000	1.000
ESR	8.8 ± 7.6	8.9 ± 7.4	8.8 ± 8.1	0.997	1.000	1.000	1.000
sACE2	0.5 ± 0.9	0.5 ± 0.7	0.3 ± 0.4	**0.029**	0.157	0.150	0.157
BVP mean	68.3 ± 32.7	103 ± 71.7	92.3 ± 49.7	0.068	0.040	0.106	0.570
BVP SD	7.5 ± 4.4	12.5 ± 9.8	9.9 ± 7.0	0.076	0.029	0.350	0.351

Abbreviations: IL—Interleukin, INF—Interferon-gamma, TNF—Tumor necrosis factor-alpha, RM-ANOVA—Repeated-measures analysis of variance, CD4/CD8 T cell Subsets (Helper/Cytotoxic), virus—virus stimulated, bacteria—bacteria stimulated, fungal—fungal stimulated, ESR—Erythrocyte sedimentation rate, sACE2—Soluble angiotensin-converting enzyme 2, BVP—Blood volume pulse amplitude. Bold means statistically significant changes.

**Table 3:** Subjective perception of stress (TICS) across time points. RM-ANOVA and post-hoc tests (adjusted by Holm-Bonferroni method).

Parameter	Measurement 1 (Mean ± SD)	Measurement 2 (Mean ± SD)	Measurement 3 (Mean ± SD)	*p*-Value Anova	M1 vs. M2	M1 vs. M3	M2 vs. M3
Work overload	15.1 ± 7.8	12.7 ± 8.3	13.7 ± 8.2	**0.009**	**0.027**	0.166	0.166
Social overload	8.7 ± 4.5	8.1 ± 4.4	7.7 ± 4.5	0.339	0.77	0.423	0.77
Pressure to succeed	12.4 ± 6.6	10.2 ± 6.7	11.3 ± 6.5	**0.018**	**0.045**	0.196	0.107
Overwhelm	6.4 ± 4.1	5.2 ± 4.3	6.2 ± 4.4	0.081	0.064	0.761	0.142
Lack of social recognition	3.9 ± 3.0	3.7 ± 2.9	4.4 ± 3.2	0.324	0.758	0.758	0.351
Social tensions	5.7 ± 2.5	5.1 ± 2.4	5.4 ± 3.1	0.533	0.594	1.000	1.000
Social isolation	5.0 ± 2.7	4.1 ± 2.7	5.4 ± 2.9	**0.049**	0.190	0.564	**0.012**
Chronic concern	5.8 ± 2.7	4.9 ± 3.1	5.6 ± 2.9	0.232	0.169	0.723	0.496

Bold means statistically significant changes.

## Data Availability

The data presented in this study are available on request from the corresponding author due to reasonable request.

## Supplementary Materials

The following supporting information can be downloaded at: https://www.mdpi.com/article/10.3390/jcm15030967/s1. Table S1: Baseline characteristics and received cryotherapy.

## Abbreviations

The following abbreviations are used in this manuscript:

WBC	Whole Body Cryotherapy
BIA	Bioelectrical Impedance Analysis
BVP	Blood Volume Pulse Amplitude
TICS	Trier Inventory for Chronic Stress
ESR	Erythrocyte Sedimentation Rate (German: BKS)
sACE2	Soluble Angiotensin Converting Enzyme 2
IL 2	Interleukin 2
IL 10	Interleukin 10
IFN γ	Interferon gamma
TNF α	Tumor Necrosis Factor alpha
CD4/CD8	T cell Subsets (Helper/Cytotoxic)
RM ANOVA	Repeated Measures Analysis of Variance
