# Supplementary material for: Whole-Body Cryotherapy at −90 °C for 9 Weeks: Effects on Immune Function, Stress, and Immune-Related and Vascular Blood Parameters in Healthy Adults—Results of an Exploratory One-Armed Pilot Study
Source: J Clin Med. 2026 Jan 25;15(3):967. doi: 10.3390/jcm15030967 (PMC12898326; doi:10.3390/jcm15030967)
Supplement: Supplementary file 1 [file jcm-15-00967-s001.zip › jcm-4074832-supplementary.pdf]

Table S1. Baseline characteristics and received cryotherapy.

Variable	N	Mean ± SD/ N(%)	Median (Q1–Q3)	Range
Age (years)	19	52.9 ± 9.8	52 (44–63)	40–71
Height (cm)	19	169.1 ± 8.2	168 (165–174)	155–186
Weight (kg)	19	68.4 ± 10.6	69 (59–77)	53–88
BMI (kg/m²)	19	23.8 ± 2.6	23 (22–25)	20–29
Sex (M/F)	19	4 / 15 (21%/79%)		
Number of cryo therapy cycles	19	18 ± 0		
Average duration (min)	19	4.5 ± 0.76	4.6 (3.9-5.2)	3.3-5.8
Total duration (min)	19	59.7 ± 6.8	59 (59-64)	38-71
Average time between sessions (d)	19	3.5 ± 0.4	3.4 (3.4-3.7)	2.2-4.1

Abbreviations: Body Mass Index (BMI)
